# Evaluation of the Age, Biomarkers, and Clinical History–Bleeding Risk Score in Patients With Atrial Fibrillation With Combined Aspirin and Anticoagulation Therapy Enrolled in the ARISTOTLE and RE-LY Trials

**DOI:** 10.1001/jamanetworkopen.2020.15943

**Published:** 2020-09-16

**Authors:** Ziad Hijazi, Jonas Oldgren, Johan Lindbäck, John H. Alexander, Marco Alings, Raffaele De Caterina, John W. Eikelboom, Michael D. Ezekowitz, Claes Held, Kurt Huber, Elaine M. Hylek, Christopher B. Granger, Renato D. Lopes, Dragos Vinereanu, Agneta Siegbahn, Lars Wallentin

**Affiliations:** 1 Department of Medical Sciences, Cardiology, Uppsala University, Uppsala, Sweden; 2 Uppsala Clinical Research Center, Uppsala University, Uppsala, Sweden; 3 Duke Clinical Research Institute, Duke Medicine, Durham, North Carolina; 4 University Medical Center Utrecht, Utrecht University, Utrecht, Netherlands; 5 University Cardiology Division, University of Pisa, Pisa, Italy; 6 Population Health Research Institute, Hamilton, Ontario, Canada; 7 Sidney Kimmel Medical College, Thomas Jefferson University, Philadelphia, Pennsylvania; 8 Cardiovascular Medicine, Lankenau Institute for Medical Research, Wynnewood, Pennsylvania; 9 Wilhelminenhospital, 3rd Department of Medicine, Cardiology and Intensive Care Medicine, Medical Faculty, Sigmund Freud University, Vienna, Austria; 10 Cardiovascular Medicine, Boston University School of Medicine, Boston, Massachusetts; 11 University and Emergency Hospital, University of Medicine and Pharmacy Carol Davila, Bucharest, Romania; 12 Clinical Chemistry, Department of Medical Sciences, Uppsala University, Uppsala, Sweden

## Abstract

**Importance:**

Most patients with atrial fibrillation (AF) and coronary artery disease have indications for preventing stroke with oral anticoagulation therapy and preventing myocardial infarction and stent thrombosis with platelet inhibition.

**Objective:**

To evaluate whether the recently developed ABC (age, biomarkers, and clinical history)–bleeding risk score might be useful to identify patients with AF with different risks of bleeding during concomitant aspirin and anticoagulation therapy.

**Design, Setting, and Participants:**

The biomarkers in the ABC-bleeding risk score (growth differentiation factor 15, hemoglobin, and troponin) were measured in blood samples collected at randomization between 2006 and 2010 in the ARISTOTLE (Apixaban for Reduction in Stroke and Other Thromboembolic Events in Atrial Fibrillation) trial and between 2005 and 2009 in the RE-LY (Randomized Evaluation of Long-term Anticoagulation Therapy) trial, both of which were multinational randomized clinical trials. The trials were reported 2011 and 2009, respectively. A total of 24 349 patients with AF (14 980 patients from the ARISTOTLE trial and 9369 patients from the RE-LY trial) were analyzed in the present cohort study. The median (interquartile range) length of follow-up was 1.8 (1.3-2.3) years in the ARISTOTLE cohort and 2.0 (1.6-2.3) years in the RE-LY cohort. Data analysis was performed from February 2018 to June 2019.

**Exposures:**

Concomitant aspirin treatment during study follow-up.

**Main Outcomes and Measures:**

Time to first occurrence of a major bleeding was determined according to International Society on Thrombosis and Hemostasis definition. Hazard ratios were estimated with Cox models adjusted for ABC-bleeding risk score and randomized treatment.

**Results:**

The median (interquartile range) age was 70 (63-76) years in the ARISTOTLE cohort and 72 (67-77) years in the RE-LY cohort (5238 patients [35.6%] in the ARISTOTLE cohort and 3086 patients [36.4%] in the RE-LY cohort were women). The total number of patients with a first major bleeding event was 651 (207 with aspirin and 444 without) in ARISTOTLE and 463 (238 with aspirin and 225 without) in RE-LY. For both cohorts, in those with a low ABC-bleeding risk score, the absolute bleeding rate was low even with concomitant aspirin treatment, whereas in those with a higher ABC-bleeding risk score, the rate of bleeding was higher with concomitant aspirin compared with oral anticoagulation alone (ARISTOTLE, hazard ratio, 1.65; 95% CI, 1.40-1.95; *P* < .001; RE-LY, hazard ratio, 1.70; 95% CI, 1.42-2.04; *P* < .001). Thus, a low annual ABC-bleeding risk (eg, 0.5% without aspirin use) would with concomitant aspirin result in an annual rate of 0.8%, and a high estimated ABC-bleeding risk (eg, 3.0%) would result in a substantially higher rate of 5.0%.

**Conclusions and Relevance:**

These findings suggest that the ABC-bleeding risk score identifies patients with different risks of bleeding when combining aspirin and oral anticoagulation. The ABC-bleeding risk score may, therefore, be a useful tool for decision support concerning intensity and duration of combination antithrombotic treatment in patients with AF and coronary artery disease.

## Key Points

**Question:**

Is the recently developed ABC (age, biomarkers, and clinical history)–bleeding risk score for patients with atrial fibrillation (AF) useful to identify patients with different risks of bleeding during concomitant aspirin and anticoagulation therapy?

**Findings:**

This cohort study examined a total of 24 349 patients with AF from 2 randomized clinical trials and found that the ABC-bleeding risk score identified patients with different risks of bleeding when combining aspirin and oral anticoagulation.

**Meaning:**

The ABC-bleeding risk score may be a useful tool for decision support concerning intensity and duration of combination antithrombotic treatment in patients with AF and coronary artery disease.

## Teaser

This cohort study evaluates whether the ABC (age, biomarkers, and clinical history)–bleeding risk score might be useful to identify patients with atrial fibrillation (AF) who have different risks of bleeding during concomitant aspirin and anticoagulation therapy.

## Introduction

In patients with atrial fibrillation (AF) and coronary artery disease (CAD), there are indications for preventing stroke with oral anticoagulation therapy and preventing myocardial infarction (MI) and stent thrombosis with platelet inhibition.^1,2^ However, as the antithrombotic therapy intensifies, the risk of major bleeding increases.^3^ This is a well-known, and rather frequently encountered, dilemma in clinical practice, because approximately 20% to 30% of patients with AF also have concomitant CAD.^4,5,6^ International guidelines^1,2^ recommend an individual risk-benefit assessment to tailor the antithrombotic treatment and its duration to an optimally balanced risk of ischemic events vs risk of bleeding. However, current bleeding risk scores provide limited guidance in the setting of AF and concomitant CAD.^5^ Recently, the ABC (age, biomarkers, and clinical history)–bleeding risk score was developed and validated in patients with AF receiving oral anticoagulation therapy for estimation of major bleeding events.^7^ The biomarker-based ABC-bleeding risk score outperformed other risk scores based on clinical variables, such as the HAS-BLED (hypertension, abnormal renal and liver function, stroke, bleeding, labile international normalized ratio, elderly, drugs or alcohol) score. Importantly, the ABC-bleeding risk score was also shown to be well-calibrated in several cohorts of patients with AF.^7,8^ The ABC-bleeding score has, therefore, been mentioned in the recent European AF and Dual Antiplatelet Therapy guidelines^1,2^ as a possible tool to estimate risk of major bleeding in patients with AF. In this cohort study, we evaluated whether the ABC-bleeding risk score might be useful to identify patients with different risks of bleeding during concomitant antiplatelet (aspirin) and anticoagulation therapy based on 9369 patients from the RE-LY (Randomized Evaluation of Long-term Anticoagulation Therapy) trial^9^ and 14 980 patients with AF from the ARISTOTLE (Apixaban for Reduction in Stroke and Other Thromboembolic Events in Atrial Fibrillation) trial.^10^ We hypothesized that the biomarker-based ABC-bleeding risk score can differentiate between the bleeding risk in patients with AF taking oral anticoagulation and concomitant aspirin.

## Methods

The ARISTOTLE trial randomized 18 201 patients with AF and an increased risk of stroke to warfarin or apixaban and was reported in 2011.^10,11^ Biomarker samples were available at baseline for 14 697 participants, with a median (interquartile range) length of follow-up of 1.8 (1.3-2.3) years. The RE-LY trial randomized 18 113 patients with AF to dabigatran or warfarin and was reported in 2009.^9,12^ Biomarker samples were available at baseline for 8468 participants, with a median (interquartile range) length of follow-up of 2.0 (1.6-2.3) years. Overall, both the ARISTOTLE and RE-LY biomarker cohorts were representative of each respective study cohort and have been described in detail elsewhere.^13,14^ Institutional review board or ethics committee approval was obtained at all sites for both trials, and all patients provided written informed consent, including consent for the biomarker substudy program. This study follows the Strengthening the Reporting of Observational Studies in Epidemiology (STROBE) reporting guideline.

### Outcome Assessment

In both the ARISTOTLE and the RE-LY trials, the primary safety outcome was major bleeding defined according to the International Society on Thrombosis and Hemostasis.^9,10^ Blinded clinical event committees reviewed and centrally adjudicated the outcome events.

### Biochemical Methods

In both the ARISTOTLE and RE-LY studies, blood samples were collected at randomization into EDTA tubes and immediately centrifuged, frozen in aliquots, and stored at −70 °C until analyzed centrally at the Uppsala Clinical Research Center laboratory in Uppsala, Sweden. High-sensitive cardiac troponin T and N-terminal prohormone of brain natriuretic peptide (NT-proBNP) were analyzed with high-sensitivity immunoassays on the Cobas Analytics e601 (Roche Diagnostics), and growth differentiation factor–15 (GDF-15) levels were detected with the Elecsys growth differentiation factor–15 precommercial assay kit P03 with the same standardization as the recently introduced routine reagent (Roche Diagnostics). All analyses were performed according to the instructions of the manufacturer and have been described elsewhere.^15,16,17^

### Statistical Analysis

All patients with no missing values for the variables included in the ABC-bleeding score (prior stroke or transient ischemic attack, age, high-sensitive cardiac troponin, and N-terminal prohormone of brain natriuretic peptide) measured at baseline were included in the analyses. The ABC-bleeding score was defined in this study as the estimated 1-year major bleeding risk.

The consistency of the ABC-bleeding score with concomitant aspirin use was assessed using a Cox regression model. The model included aspirin use and the estimated 1-year ABC-bleeding risk as a restricted cubic spline, with 4 knots placed at the 5th, 35th, 65th, and 95th sample percentiles, and the interaction between aspirin status and the linear part of the ABC-bleeding risk. If the test of the interaction was not significant, a model excluding the interaction term was fitted to estimate the relative hazard of bleeding according to aspirin use. The fitted model was also used to estimate the absolute increase in bleeding for 2 examples of low (0.5%) and high (3.0%) ABC-bleeding risks without aspirin. These examples were chosen because there are no established cutoffs for low or high annual major bleeding risks in patients with AF; previously, risks below 1% and above 2% have been indicated, although not established.^18^

In the ARISTOTLE trial, aspirin use was recorded continuously during follow-up and was therefore included as a time-updated covariate in the model. In the RE-LY trial, a patient was assumed to be taking aspirin if she or he was taking aspirin at any time during follow-up. The models did not include any further adjustment for confounders because the ABC-bleeding risk score already incorporates the main risk factors for major bleeding and further adjustment would not be expected to improve the model.^7^

Nevertheless, in a sensitivity analysis of the ARISTOTLE data, a Cox regression model including potential baseline and time-updated confounders was fitted. Baseline variables included age, sex, diabetes, hypertension, history of MI, history of peripheral arterial disease, and history of vascular disease. Postrandomization variables included whether the patient was currently taking the study drug, MI, angina, percutaneous coronary intervention, coronary stenting, nonmajor bleeding and interactions between prior MI and nonmajor bleeding, MI and nonmajor bleeding, study drug and angina, study drug and percutaneous coronary intervention, and nonmajor bleeding. The results were materially unaltered compared with the unadjusted model; therefore, we only present the results from the simpler model. Furthermore, the 3-way interaction between the ABC-bleeding risk score, aspirin use, and study treatment was evaluated by fitting a Cox model including the ABC-bleeding risk score, a treatment combination variable (aspirin or no aspirin in combination with warfarin or apixaban in ARISTOTLE, and warfarin or dabigatran [110 mg or 150 mg] in RE-LY) and the interaction between the ABC-bleeding risk score and the treatment combination variable.

Results from all models are presented graphically as plots of hazard ratios (HRs) with an arbitrary reference point set to 2.0% 1-year ABC-bleeding risk in the no-aspirin group. The ABC-bleeding and HAS-BLED risk scores were calculated according to previously described methods.^7^

All tests were 2-sided, with *P* < .05 denoting statistical significance. All analyses were done using R statistical software version 3.6.1 (R Project for Statistical Computing). Data analysis was performed from February 2018 to June 2019.

## Results

### Baseline Characteristics

A total of 24 349 patients with AF were analyzed in the present cohort study. The median (interquartile range) age was 70 (63-76) years in the ARISTOTLE cohort and 72 (67-77) years in the RE-LY cohort; 5238 patients (35.6%) in the ARISTOTLE cohort and 3086 patients (36.4%) in the RE-LY cohort were women. The characteristics according to concomitant aspirin use or no aspirin use are shown in Table 1 for the ARISTOTLE and RE-LY cohort. The proportion of patients taking concomitant aspirin was 31.0% (4638 patients) at day 1 in the ARISTOTLE cohort and approximately 20% on any day after day 1.^4^ In the RE-LY cohort, the proportion of patients taking concomitant aspirin at any time during follow-up was 36.4% (3413 patients). In both cohorts, a higher proportion of patients receiving concomitant aspirin had vascular disease (eg, prior MI, CAD, or peripheral artery disease). The presence of diabetes was also more common in patients with concomitant aspirin. Patients with prior vitamin K antagonist use were more common in the group without concomitant aspirin.

**Table 1.** Baseline Characteristics in the ARISTOTLE Cohort by Aspirin Status on Day 1 (Day of Randomization) and in the RE-LY Cohort by Aspirin Status During Follow-up, Anytime During the Study, Including Biomarkers Used in the ABC-Bleeding Score

Variable	Patients, No. (%)			
	ARISTOTLE		RE-LY	
	No aspirin (n = 10 342)	Aspirin (n = 4638)	No aspirin (n = 5956)	Aspirin (n = 3413)
Randomized treatment: warfarin	5193 (50.2)	2289 (49.4)	1985 (33.3)	1144 (33.5)
Received dabigatran				
110 mg	NA	NA	1972 (33.1)	1137 (33.3)
150 mg	NA	NA	1999 (33.6)	1132 (33.2)
Age, y, median (IQR)	70.0 (62.0-76.0)	70.0 (63.0-76.0)	72.0 (67.0-77.0)	72.0 (67.0-78.0)
Female	3769 (36.4)	1562 (33.7)	2275 (38.2)	1133 (33.2)
Body mass index, median (IQR)^a^	28.5 (25.3-32.5)	28.6 (25.3-32.8)	28.0 (25.1-31.6)	27.9 (25.1-31.2)
Missing values, No.	49	22	3	4
Systolic blood pressure, mm Hg, median (IQR)	130.0 (120.0-140.0)	130.0 (120.0-140.0)	130.0 (120.0-142.0)	130.0 (120.0-140.0)
Missing values, No.	25	8	8	5
Diabetes	2454 (23.7)	1243 (26.8)	1196 (20.1)	883 (25.9)
Hypertension	8961 (86.6)	4153 (89.5)	4660 (78.2)	2731 (80.0)
Current smoker	870 (8.4)	349 (7.5)	467 (7.8)	254 (7.4)
Missing values, No.	12	2		
Alcohol	264 (2.6)	114 (2.5)	866 (14.5)	456 (13.4)
Permanent or persistent atrial fibrillation	8865 (85.7)	3846 (82.9)	4255 (71.5)	2078 (60.9)
Missing values, No.	3	0	2	2
Prior stroke or transient ischemic attack	1948 (18.8)	861 (18.6)	1159 (19.5)	664 (19.5)
Prior bleeding	1701 (16.4)	736 (15.9)	678 (11.4)	505 (14.8)
Anemia	665 (6.4)	342 (7.4)	678 (11.4)	505 (14.8)
Missing values, No.	6	5	0	0
Heart failure	3115 (30.1)	1536 (33.1)	1685 (28.3)	1026 (30.1)
Missing values, No.	0	0	1	0
Prior myocardial infarction	1008 (9.7)	918 (19.8)	747 (12.5)	842 (24.7)
Missing values, No.	1	0	0	0
Prior peripheral arterial disease	456 (4.4)	274 (5.9)	186 (3.1)	159 (4.7)
Missing values, No.	1	0	1	0
Prior vascular disease	2028 (19.6)	1695 (36.5)	882 (14.8)	937 (27.5)
Warfarin within 7 d of randomization	6455 (62.5)	1582 (34.2)	4397 (73.8)	1609 (47.1)
Missing values, No.	18	11	0	0
Estimated glomerular filtration rate, mL/min, median (IQR)	74.5 (57.0-96.0)	73.1 (56.1-93.8)	65.5 (54.5-77.4)	63.7 (52.7-76.4)
Missing values, No.	37	15	68	23
Growth differentiation factor 15, ng/L, median (IQR)	1348.0 (965.0-1992.0)	1464.0 (1005.0-2216.0)	1476.0 (1092.0-2124.0)	1592.0 (1142.2-2338.0)
Missing values, No.	117	65	467	251
High-sensitive cardiac troponin T, ng/L, median (IQR)	10.7 (7.4-16.3)	11.6 (7.8-17.6)	11.7 (7.5-18.8)	13.1 (8.2-20.6)
Missing values, No.	49	34	417	233
Hemoglobin, g/dL, median (IQR)	14.3 (13.2-15.3)	14.2 (13.1-15.3)	14.3 (13.3-15.3)	14.2 (13.1-15.3)
Missing values, No.	53	17	100	59

Abbreviations: ABC, age, biomarkers, and clinical history; ARISTOTLE, Apixaban for Reduction in Stroke and Other Thromboembolic Events in Atrial Fibrillation; IQR, interquartile range; NA, not applicable; RE-LY, Randomized Evaluation of Long-term Anticoagulation Therapy.

SI conversion factor: To convert hemoglobin to grams per liter, multiply by 10.0.

^a^ Body mass index is calculated as weight in kilograms divided by height in meters squared.

### Risk of Major Bleeding With Concomitant Aspirin Use and ABC-Bleeding Risk Score

#### ARISTOTLE Cohort

**Figure 1.**
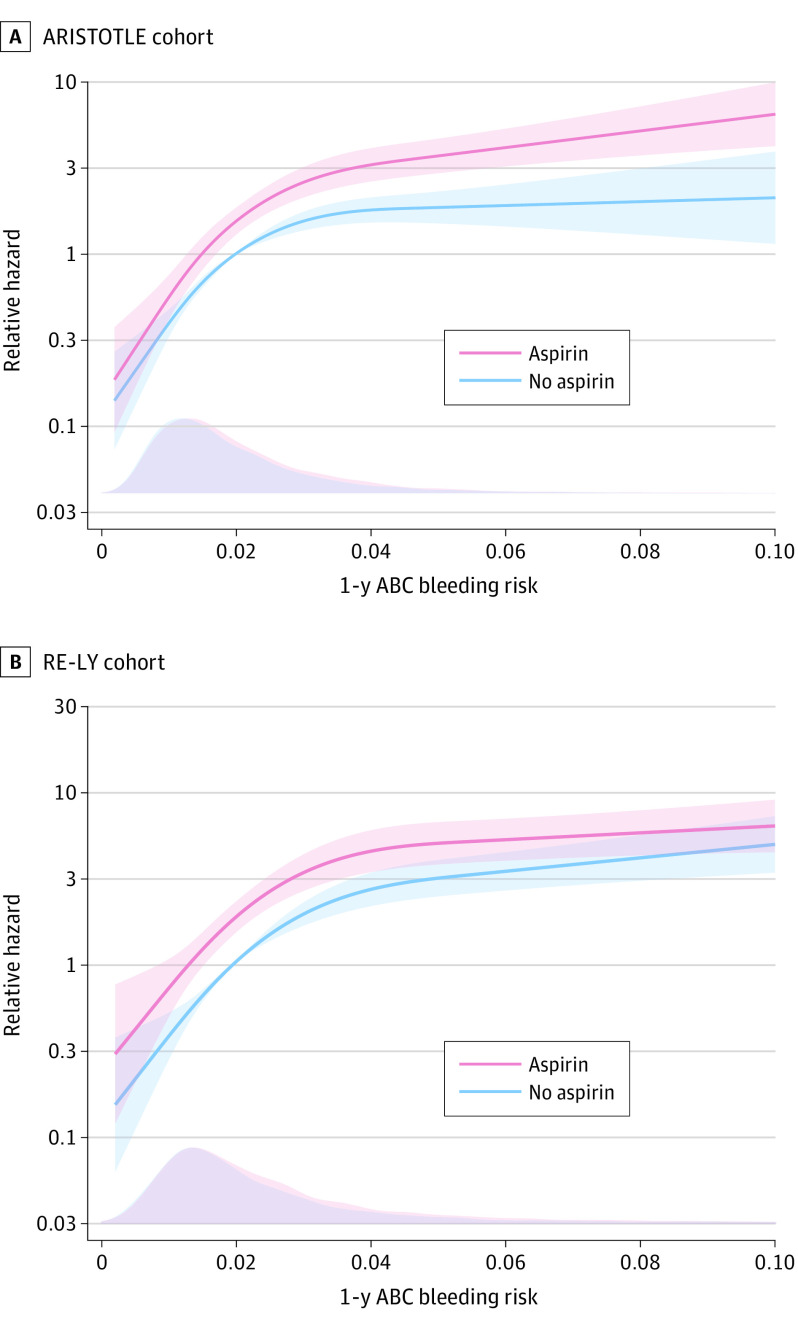
Relative Hazard of Major Bleeding Comparing Patients With Oral Anticoagulation Taking Aspirin With Patients Not Taking Aspirin by Estimated 1-Year Risk According to the ABC (Age, Biomarkers, and Clinical History)–Bleeding Risk Score Graphs show relative hazards (lines) and 95% CIs (shaded areas) for the ARISTOTLE (Apixaban for Reduction in Stroke and Other Thromboembolic Events in Atrial Fibrillation) (A) and RE-LY (Randomized Evaluation of Long-term Anticoagulation Therapy) (B) cohorts. The bottom of each panel shows the distribution of the patients with ABC-bleeding risk scores as a density plot for each group at baseline.

The total number of patients with a first major bleeding event was 651 (207 while taking aspirin and 444 while not taking aspirin) during 24 903 person-years of follow-up. The annual rate of major bleeding during follow-up was higher in patients taking aspirin than in those not taking aspirin (4.04% vs 2.24%) (Table 2). The bottom quarter of the patients had an estimated ABC-bleeding risk less than 1.1% annually. In these patients, the absolute bleeding risk was low even with concomitant aspirin treatment (Figure 1A). In those in the top quarter with an ABC-bleeding risk greater than 2.5%, the absolute risk of bleeding was higher with concomitant aspirin compared with oral anticoagulation alone (Figure 1A). The *P* value for the test of the null hypothesis of no multiplicative interaction was .07. In a model without the interaction term, adjusted for ABC-bleeding risk score and randomized treatment, concomitant aspirin treatment increased bleeding significantly (HR, 1.65; 95% CI, 1.40-1.95; *P* < .001). Thus, a low ABC-bleeding risk score without aspirin (eg, 0.5% annually) would with concomitant aspirin result in an annual rate of 0.8%, and a high estimated ABC-bleeding risk score without aspirin (eg, 3.0% annually) would result in a substantially higher absolute annual rate for major bleeding of 5.0%.

**Table 2.** Event Rates of Major Bleeding

Group^a^	Patients, No.	Events, No.	Person-years, No.	Incidence rate/100 person-years (95% CI)
ARISTOTLE				
No aspirin	11 943	444	19 781	2.24 (2.04-2.46)
Aspirin	4987	207	5122	4.04 (3.51-4.63)
Total	14 697	651	24 903	2.61 (2.42-2.82)
RE-LY				
No aspirin	5372	225	10 519	2.14 (1.87-2.44)
Aspirin	3096	238	5693	4.18 (3.67-4.75)
Total	8468	463	16 212	2.86 (2.60-3.13)

Abbreviations: ARISTOTLE, Apixaban for Reduction in Stroke and Other Thromboembolic Events in Atrial Fibrillation; RE-LY, Randomized Evaluation of Long-term Anticoagulation Therapy.

^a^ ARISTOTLE data are based on time-updated aspirin data. RE-LY data are based on aspirin data from anytime during the study.

**Figure 2.**
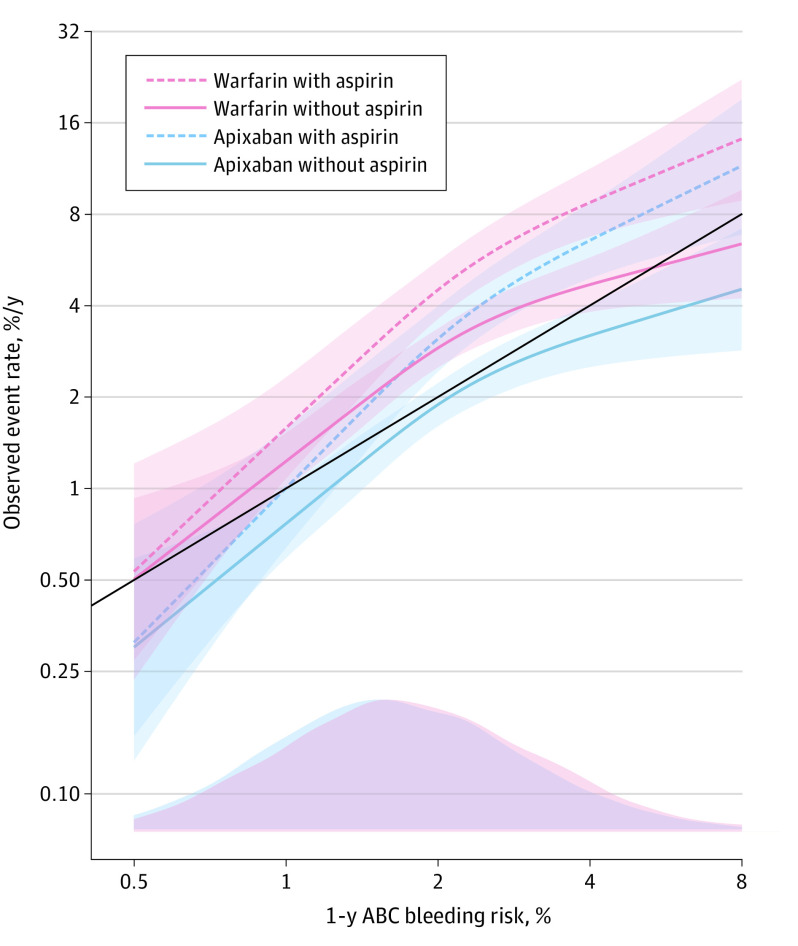
One-Year Risk of Major Bleeding Rate as a Function of the ABC (Age, Biomarkers, and Clinical History)–Bleeding Score by Different Treatment Combinations Lines denote hazard ratios. Shaded areas denote 95% CIs. The solid black line indicates perfect calibration. The bottom of the graph shows the distribution of the patients with ABC-bleeding risk scores as a density plot for each group at baseline.

The observed event rate for major bleeding as a function of the ABC-bleeding score by the different combinations of antithrombotic treatment is shown in Figure 2. At low estimated bleeding risk with the ABC-bleeding score, the model showed good calibration. With higher estimated bleeding risk, concomitant use of aspirin conferred higher than estimated bleeding rates.

#### RE-LY Cohort

The total number of patients with first major bleeding events was 463 (238 with aspirin and 225 without) during 16 212 person-years of follow-up. The annual rate of major bleeding during follow-up was higher in patients with aspirin than without aspirin (4.18% vs 2.14%) (Table 2). Overall, the results in the RE-LY cohort were similar to those of the ARISTOTLE cohort. The bottom quarter of the patients had an estimated ABC-bleeding risk less than 1.3%, whereas the top quarter had an ABC-bleeding risk greater than 2.8%. The relative hazard of major bleeding associated with aspirin use was similar across the full range of the ABC-bleeding risk score (Figure 1B; *P* for interaction = 0.2). In Cox models adjusted for ABC-bleeding risk score and randomized treatment, concomitant aspirin treatment increased bleeding significantly (HR, 1.70; 95% CI, 1.42-2.04; *P* < .001). Thus, a low ABC-bleeding risk score without aspirin (eg, 0.5% annually) would with concomitant aspirin result in an annual rate of 0.85%, and a high estimated ABC-bleeding risk score without aspirin, (eg, 3.0% annually) would result in a substantially higher annual rate for major bleeding of 5.1%.

### Sensitivity Analyses According to Different Oral Anticoagulant Therapies

**Figure 3.**
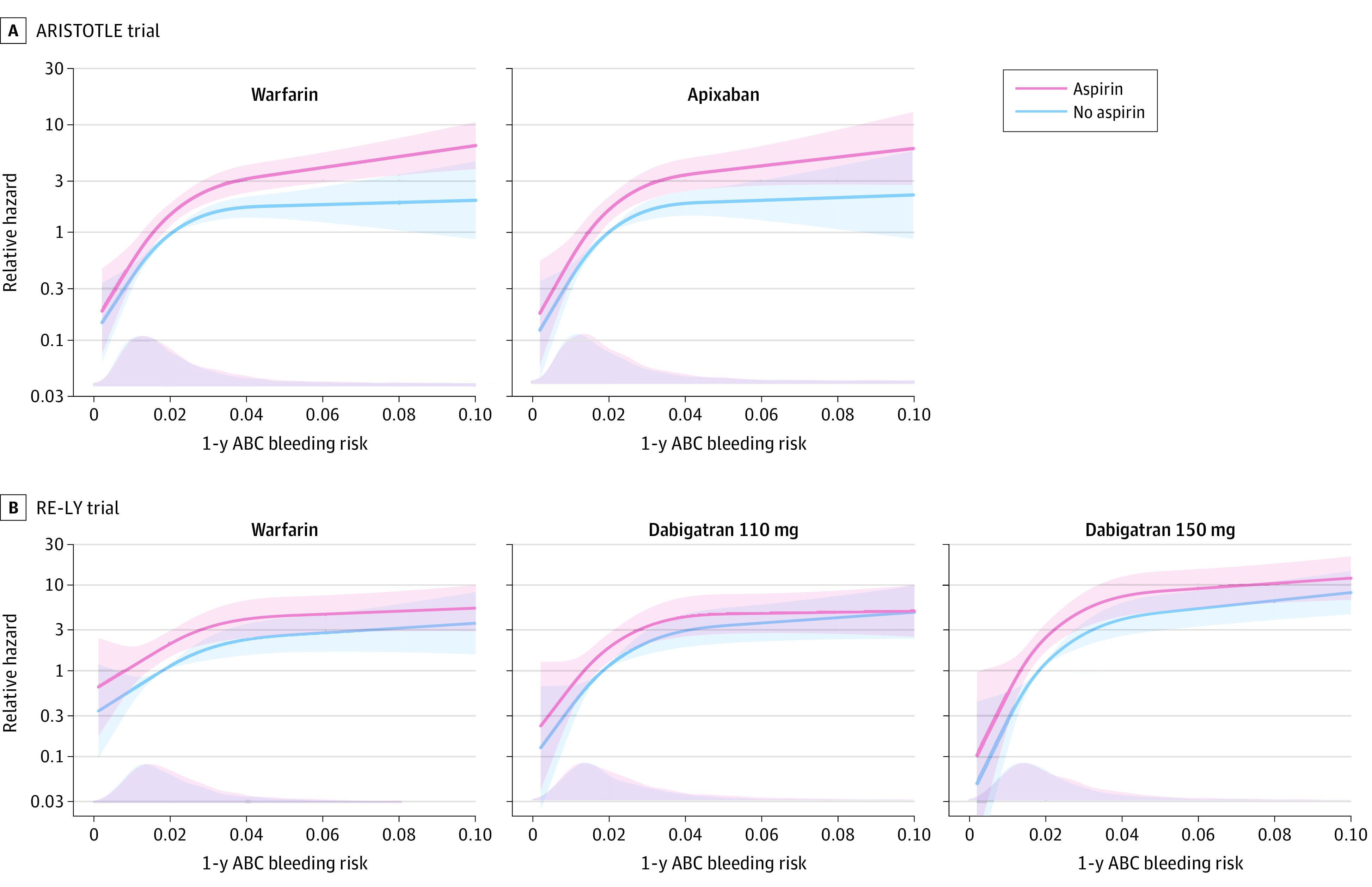
Relative Risk of Major Bleeding by Study Treatment Graphs show data for the ARISTOTLE (Apixaban for Reduction in Stroke and Other Thromboembolic Events in Atrial Fibrillation) (A) and RE-LY (Randomized Evaluation of Long-term Anticoagulation Therapy) (B) cohorts. Lines denote hazard ratios. Shaded areas denote 95% CIs. The bottom of each panel shows the distribution of the patients ABC (age, biomarkers, and clinical history)–bleeding risk scores as a density plot for each group at baseline.

Although limited by a smaller number of events, sensitivity analyses were performed in relation to different oral anticoagulant therapies. The results were similar between the ARISTOTLE cohort (warfarin, HR, 1.62 [95% CI, 1.30-2.02]; apixaban, HR, 1.69 [95% CI, 1.32-2.18]) (Figure 3A) and the RE-LY cohort (warfarin, HR, 1.77 [95% CI, 1.29-2.42]; dabigatran 110 mg, HR, 1.50 [95% CI, 1.08-2.07]; dabigatran 150 mg, HR, 1.84 [95% CI, 1.34-2.52]) (Figure 3B).

### Comparison With the HAS-BLED Score and Discriminatory Value

The risk of major bleeding according to the HAS-BLED score in patients with oral anticoagulation taking aspirin compared with those not taking aspirin are shown in eFigure in the Supplement. The discriminatory ability to estimate the risk of major bleeding in patients with AF treated with oral anticoagulation and concomitant aspirin was 0.62 and 0.63 for the HAS-BLED score in the ARISTOTLE and RE-LY cohorts, and 0.68 and 0.72 for the ABC-bleeding score, respectively. Additionally, the information gained from these risk scores was also assessed in models containing both scores (eTable in the Supplement). This showed that the ABC-bleeding risk score remained significant in the model, whereas the HAS-BLED score was no longer significant (eFigure in the Supplement).

## Discussion

The main findings in this study are that the ABC-bleeding risk score is able to stratify patients with AF receiving the combination of aspirin and oral anticoagulation according to their risk of bleeding. A low estimated risk of major bleeding according to the ABC-bleeding risk score showed a low bleeding risk even with concomitant aspirin treatment, whereas a higher ABC-bleeding risk score was associated with higher bleeding risk. These findings suggest that the ABC-bleeding risk score may be a useful tool for decision support concerning intensity and duration of combination antithrombotic treatment in patients with AF and CAD to improve the risk-benefit balance with different antithrombotic strategies.

The added value of biomarkers for improved risk estimation in patients with AF have so far been rather consistent in several cohorts, including randomized clinical trial cohorts, as well as less selected observational registry cohorts.^19,20,21^ The ABC-bleeding risk score, which includes the most relevant variables, including both biomarkers and clinical data, was developed and validated using robust methods and very large cohorts of patients with AF.^7,22,23^ The ABC-bleeding risk score outperforms traditional risk scores for stroke, death, and major bleeding, in comparison with risk scores based on clinical variables. In addition, the ABC-bleeding risk score has shown to be well calibrated, with estimated risk aligning well with observed risk during follow-up.^7,22,23^ Biomarkers and the ABC-bleeding risk scores in patients with AF have, therefore, been suggested as potential new tools for improved risk estimation in the latest European guidelines for management of AF.^1^ Similarly, the European Dual Antiplatelet Therapy guidelines refer to, among others, the ABC-bleeding risk score as a possible tool to estimate bleeding risk in patients with AF with an indication for added antiplatelet therapy to assess the bleeding risk and make informed decisions regarding the intensity and duration of concomitant antiplatelet therapy.^2^ Patients with AF and CAD considered to have a high ischemic risk (eg, because of acute coronary syndrome or other anatomical or procedural characteristics) should, according to the guidelines, be considered for a more intense and prolonged antithrombotic strategy. If these patients are considered to have a high risk of bleeding, a less intense and shorter duration of concomitant antiplatelet therapy is suggested in the guidelines. To our knowledge, the present study is the first to evaluate the ABC-bleeding risk score in patients with AF and chronic CAD receiving concomitant single-antiplatelet therapy vs oral anticoagulation, and our findings support that the ABC-bleeding risk score is useful for estimating bleeding risk in such patients.

The landmark non–vitamin K antagonist oral anticoagulant (NOAC) trials^24^ have showed that patients taking NOACs have similar or lower risks of major bleeding in comparison with patients taking warfarin. Furthermore, substudies from these NOAC trials have shown that the risk of major bleeding increases with concomitant use of aspirin, although with preserved safety with NOACs compared with warfarin; for example, even if the risk of major bleeding increases with concomitant antiplatelet therapy in both the NOAC as well as the warfarin groups, the relative risk is still lower with NOACs.^4,25,26,27^ Nonetheless, in some patients, the risk of ischemic events may warrant a more aggressive antithrombotic treatment strategy. Recent randomized clinical trials have repeatedly highlighted the changing balance between benefits and risks with increasing intensity of antithrombotic treatment. For instance, the COMPASS trial,^28^ which compared the use of dual therapy (low-dose rivaroxaban in combination with aspirin) with single-antithrombotic therapy (aspirin alone) in patients with stable CAD without AF, found that the combination of low-dose rivaroxaban with aspirin significantly reduced the risk of ischemic cardiovascular events. However, this comes at a cost of increased risk of major bleeding events. A similar pattern is also evident early after coronary events (ie, percutaneous coronary interventions and/or acute coronary syndromes) when comparing triple- and dual-antithrombotic therapy strategies in patients with AF.^29,30^ These studies consistently show that the risk of clinically relevant, as well as major, bleeding events are substantially lower with less-aggressive antithrombotic treatment strategies (ie, dual- vs triple-antithrombotic therapy).^29,30^ However, this might, to varying degrees, come at the price of an increased risk of ischemic events, including stent thrombosis.^29,30,31^ Risk score models that improve the risk stratification concerning bleeding events would thus be useful to better identify patients for whom more intensive antithrombotic therapy is suitable. By providing an individualized treatment strategy, these models would also improve patient outcomes.

In the present study, we expanded on previous findings by showing that the biomarker-based ABC-bleeding risk score successfully identifies patients with different risks of bleeding when combining single-antiplatelet therapy with aspirin and oral anticoagulation. The ABC-bleeding risk score may, therefore, be a useful tool for decision support concerning the intensity and duration of combination antithrombotic treatment in patients with AF and stable CAD. However, it is important to keep in mind that concomitant use of aspirin will offset the calibration of the ABC-bleeding score, because the observed risk of bleeding will increase by adding concomitant antiplatelet therapy.

### Limitations

This study has limitations that should be addressed. The patients in the ARISTOTLE trial were clinically stable, and the biomarkers used in the ABC-bleeding risk score, in particular troponin, will be affected in patients with acute coronary syndromes. Thus, the ABC-bleeding risk score should preferably be assessed at a later time point, such as during routine outpatient postdischarge check-up, for the most accurate ABC-bleeding risk estimation. Because antiplatelet therapy was not randomized, there may be confounding by indication, and thus evaluation of ischemic risks was not performed. For the ARISTOTLE data, it was possible to use an advanced statistical model in which aspirin was used as a time-updated covariable. In the RE-LY cohort, the status of concomitant antiplatelet therapy was defined as any time during the study and may not completely reflect the medication during study follow-up. Nevertheless, the results were very consistent between the 2 cohorts. Furthermore, as with every risk score, a prospective evaluation of the risk score such as the ongoing ABC-AF study (ClinicalTrials.gov identifier NCT03753490) is desirable to fully assess the clinical usefulness of the ABC-bleeding score for better tailoring of antithrombotic treatment strategies.

## Conclusions

The ABC-bleeding risk score accurately identifies patients with different risks of bleeding when combining aspirin and oral anticoagulation; low ABC-bleeding risk scores were associated with low bleeding risk, and higher ABC-bleeding risk scores were associated with higher bleeding risk. These data suggest that the ABC-bleeding risk score may provide useful information concerning intensity and duration of combination antithrombotic treatment in patients with AF and stable CAD.

## Notes

Supplement. **eFigure.** Relative Hazard of Major Bleeding Comparing Patients With Oral Anticoagulation on Aspirin (ASA) With Patients Not on ASA in ARISTOTLE and RE-LY by Estimated HAS-BLED Risk **eTable. **ANOVA Table for the Cox Regression Model Including Aspirin, the ABC-Bleeding and HAS-BLED Scores, and the Interaction Between Aspirin and the Respective Bleeding Risk Scores
