# Supplementary material for: Evaluation of the Age, Biomarkers, and Clinical History–Bleeding Risk Score in Patients With Atrial Fibrillation With Combined Aspirin and Anticoagulation Therapy Enrolled in the ARISTOTLE and RE-LY Trials
Source: JAMA Netw Open. 2020 Sep 16;3(9):e2015943. doi: 10.1001/jamanetworkopen.2020.15943 (PMC7495236; doi:10.1001/jamanetworkopen.2020.15943)
Supplement: Supplement. — eFigure. Relative Hazard of Major Bleeding Comparing Patients With Oral Anticoagulation on Aspirin (ASA) With Patients Not on ASA in ARISTOTLE and RE-LY by Estimated HAS-BLED Risk eTable. ANOVA Table for the Cox Regression Model Including Aspirin, the ABC-Bleeding and HAS-BLED Scores, and the Interaction Between Aspirin and the Respective Bleeding Risk Scores [file jamanetwopen-e2015943-s001.pdf]

Supplementary Online Content

Hijazi Z, Oldgren J, Lindbäck J, et al. Evaluation of the age, biomarkers, and clinical history–bleeding risk score in patients with atrial fibrillation with combined aspirin and anticoagulation therapy enrolled in the ARISTOTLE and RE-LY trials. *JAMA Netw Open*. 2020;3(9):e2015943. doi:10.1001/jamanetworkopen.2020.15943

eFigure. Relative Hazard of Major Bleeding Comparing Patients With Oral Anticoagulation on Aspirin (ASA) With Patients Not on ASA in ARISTOTLE and RE-LY by Estimated HAS-BLED Risk
eTable. ANOVA Table for the Cox Regression Model Including Aspirin, the ABC-Bleeding and HAS-BLED Scores, and the Interaction Between Aspirin and the Respective Bleeding Risk Scores

This supplementary material has been provided by the authors to give readers additional information about their work.

© 2020 Hijazi Z et al. *JAMA Network Open.*

eFigure. Relative Hazard of Major Bleeding Comparing Patients With Oral Anticoagulation on Aspirin (ASA) With Patients Not on ASA in ARISTOTLE and RE-LY by Estimated HAS-BLED Risk

A. The relative hazard of major bleeding comparing patients with oral anticoagulation on aspirin (ASA) with patients not on ASA in ARISTOTLE by estimated HAS-BLED risk

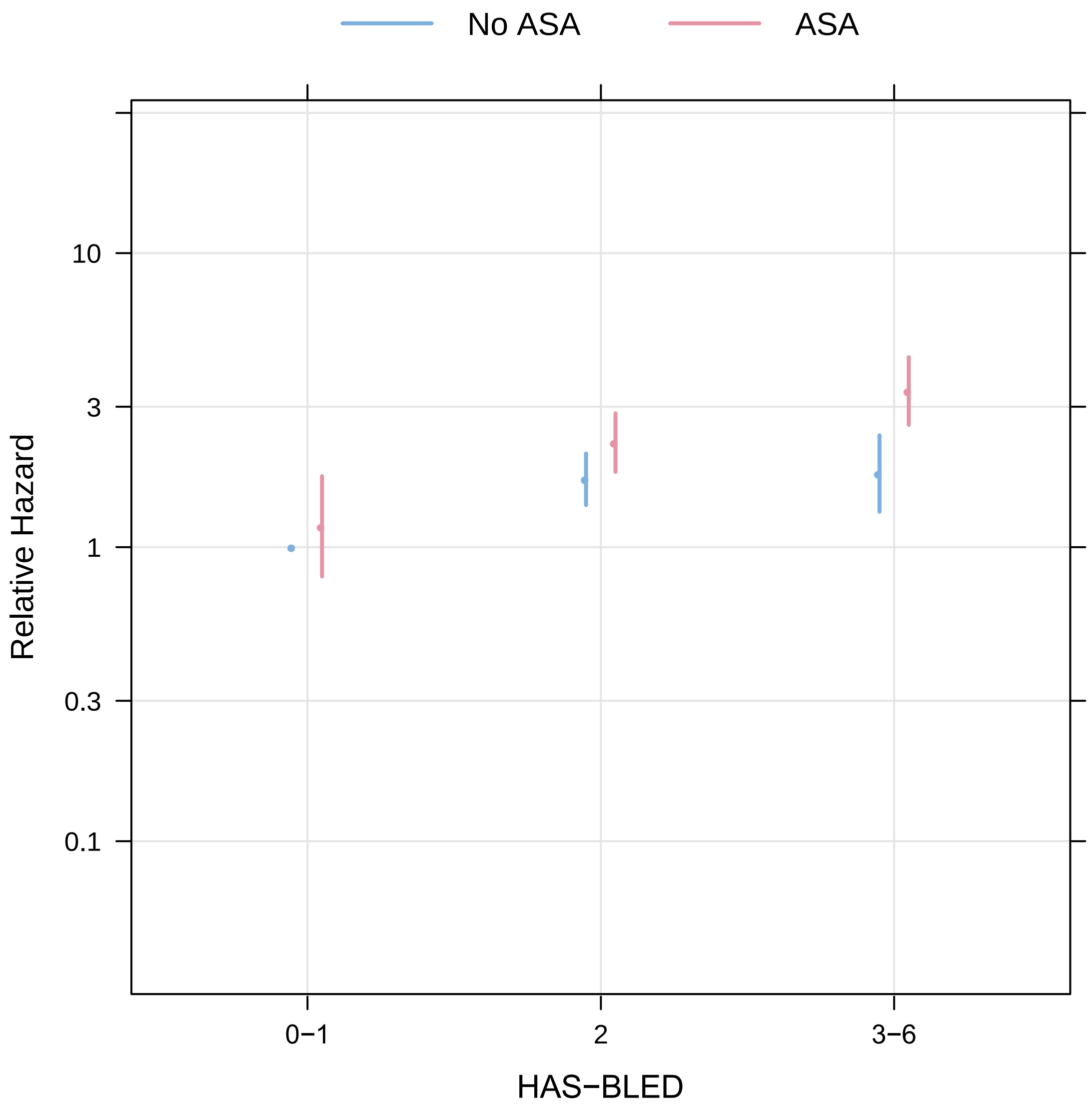

© 2020 Hijazi Z et al. *JAMA Network Open.*

B. The relative hazard of major bleeding comparing patients with oral anticoagulation on aspirin (ASA) with patients not on ASA in RE-LY by estimated HAS-BLED risk

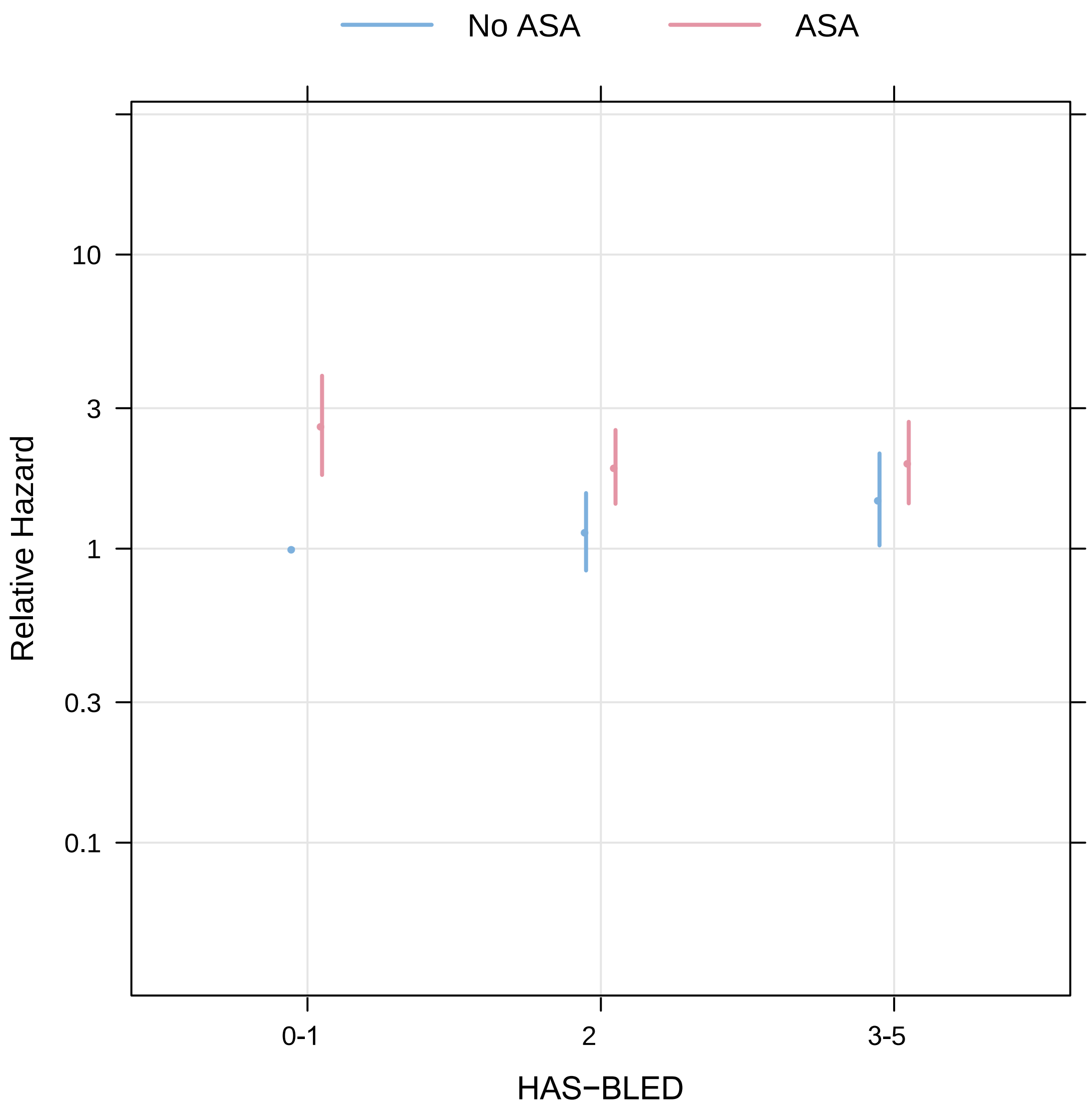

© 2020 Hijazi Z et al. *JAMA Network Open.*

eTable. ANOVA Table for the Cox Regression Model Including Aspirin, the ABC-Bleeding and HAS-BLED Scores, and the Interaction Between Aspirin and the Respective Bleeding Risk Scores

A. ARISTOTLE

Factor	Chi-Square	d.f.	*P*
On aspirin (Factor+Higher Order Factors)	26.41	3	<.0001
All Interactions	3.67	2	0.1596
ABC-bleeding (Factor+Higher Order Factors)	177.22	4	<.0001
All Interactions	3.57	1	0.0590
Nonlinear	66.87	2	<.0001
HAS-BLED (Factor+Higher Order Factors)	4.51	2	0.1047
All Interactions	0.03	1	0.8726
On aspirin * ABC-bleeding (Factor+Higher Order Factors)	3.57	1	0.0590
On aspirin * HAS-BLED (Factor+Higher Order Factors)	0.03	1	0.8726
TOTAL INTERACTION	3.67	2	0.1596
TOTAL NONLINEAR + INTERACTION	68.45	4	<.0001
TOTAL	289.47	7	<.0001

B. RE-LY:

Factor	Chi-Square	d.f.	*P*
Aspirin (Factor+Higher Order Factors)	35.32	4	<.0001
All Interactions	8.42	3	0.0381
HAS-BLED (Factor+Higher Order Factors)	7.33	4	0.1195
All Interactions	6.30	2	0.0430
ABC-bleeding (Factor+Higher Order Factors)	186.79	4	<.0001
All Interactions	0.51	1	0.4754
Nonlinear	86.26	2	<.0001
Aspirin * ABC-bleeding (Factor+Higher Order Factors)	0.51	1	0.4754
Aspirin * HAS-BLED (Factor+Higher Order Factors)	6.30	2	0.0430
TOTAL INTERACTION	8.42	3	0.0381
TOTAL NONLINEAR + INTERACTION	93.38	5	<.0001
TOTAL	266.31	9	<.0001

© 2020 Hijazi Z et al. *JAMA Network Open.*
